# Pedagogical Strategies for Teaching Conceptual Models and Nursing Theories in Undergraduate Students: A Scoping Review

**DOI:** 10.1111/jan.16888

**Published:** 2025-04-01

**Authors:** Aurélie Demagny‐Warmoes, Marvin Duchadeau, Paul Quindroit, Sébastien Colson

**Affiliations:** ^1^ IFMS‐Valenciennes Hospital Valenciennes France; ^2^ Aix‐Marseille University EA 3279‐CEReSS Marseille France; ^3^ Faculty of Medical and Paramedical Sciences, Nursing School, CEReSS, APHM Aix‐Marseille Université Marseille France; ^4^ Univ. Lille, CHU Lille, ULR 2694—METRICS: Évaluation Des Technologies de Santé et Des Pratiques Médicales Lille France

**Keywords:** baccalaureate nursing education, nursing science, nursing theory, scoping review, teaching methods

## Abstract

**Background:**

Teaching nursing theories is essential to structure and guide clinical practice, yet their integration into initial training courses remains a challenge.

**Aim:**

To map the educational practices used to teach nursing theories and conceptual models in initial nursing training programmes.

**Design:**

A scoping review.

**Methods:**

Is review was conducted following the guidelines of the Joanna Briggs Institute and the PRISMA‐ScR. A comprehensive search of five databases and in the grey literature was conducted to find studies from the inception of the databases to January 2024. A total of 32 articles meeting the inclusion criteria were selected for analysis.

**Results:**

The findings reveal a wide variety of educational techniques, ranging from traditional lectures to innovative Methods such as simulation and virtual games. The analysis also shows that these educational practices cover a broad Spectrum of theories, from well‐established theories that have stood the test of time, such as watson's, to more recent Theories like the fundamentals of care.

**Conclusion:**

While integrating nursing theories into initial training programmes is crucial, further research is needed to assess the effectiveness of the pedagogical strategies used to teach them. The mapping of educational practices carried out in this review serves as a valuable resource for educators, providing a structured basis to diversify and enrich the teaching of nursing theories.

**Implications for Practice:**

This scoping review explores for the first time the range of pedagogical practices used in the teaching of nursing theories on an international scale. It provides a valuable resource for educators, allowing them to enrich their pedagogical approach. It offers a structured overview of the various possible methods, thus facilitating the adaptation of teaching strategies to different contexts. This methodological diversity can also serve as a source of inspiration for developing new concepts for teaching disciplinary fundamentals. The renewed interest in teaching disciplinary fundamentals underscores the relevance of this review in the current context.

**Impact:**

Through this mapping of pedagogical strategies, this scoping review contributes to improving the quality of teaching nursing disciplinary fundamentals internationally. The results provide a foundation for developing specific courses on nursing conceptual models and theories, offering educators various possibilities to enrich their teaching methods while adapting to local contexts and student needs.

**Patient or Public Contribution:**

No patient or public contribution. This is a scoping review.

**Trial Registration:**

https://osf.io/gj35n

**Summary**

- What already is know?
  ○ Integrating nursing theories into initial training remains a persistent challenge.
  ○ Teaching nursing theories is essential from the beginning of nursing curricula.
  ○ Disciplinary fundamentals structure clinical practice.
  ○ Nursing theories promote the development of a strong professional identity among students.
  ○ Nursing theories support structured clinical reasoning.
- What this papper adds?
  ○ Highlights diverse teaching methods, from traditional lectures to innovatives approaches.
  ○ Demonstrates significant creativity among nursing educators.
  ○ Shows that nursing theories and conceptuals models are still relevant and not outdated.
  ○ Confirms contempory theories, such as Fundamentals of Care, are included in teaching.
  ○ Illustrates the importance of combining disciplinary heritage with recent theoretical developments in education programmes.
  ○ Identifies the primary current challenge as assessing the effectiveness of these pedagogical strategies.
  ○ Suggests the need to optimize these strategies to better integrate nursing theories into teaching.

## 1 Introduction

Since the second half of the 20th century, nursing science has built a substantial body of scientific knowledge organised around conceptual models and theories developed through research (Alligood 2021). These foundational elements, which constitute the core of the discipline, are essential for structuring and guiding nursing practice. Conceptual models and nursing theories provide explanatory frameworks for understanding health phenomena, informing clinical reasoning, promoting health and implementing scientifically based interventions (Meleis 2017). These theories also serve as evidence of the ongoing evolution of disciplinary knowledge, contributing to the development of an ontological perspective unique to nursing and, consequently, to a professional nursing identity (Jenkins et al. 2021). Based on this premise, the use of theoretical frameworks from nursing science in education becomes essential for valuing nursing knowledge and demonstrating nursing's unique contribution to the quality of care, especially in an interprofessional context (Smith 2019). When applied effectively, a theoretical framework can enhance the quality of nursing care, with measurable effects on patient health outcomes (Conn et al. 2001). Conversely, the absence of theoretical references may lead to less effective interventions, lacking a clear rationale for their impact (Conn et al. 2001). Nevertheless, despite the wealth of available nursing knowledge, reports often indicate a minimal use of nursing theories to guide care practice, favouring disciplinary borrowings from other fields (Kitson 2018).

Although the importance of these theories is recognised, teaching them, especially in contexts where the discipline is still developing, remains a major challenge (Jovic et al. 2014). The abstract nature of theoretical concepts can make them difficult to teach, particularly to beginning students who may perceive them as complex (Younas and Parsons 2019). The gap between taught theories and their application in clinical practice is well documented (Lindell 2019). This gap risks hindering students' ability to utilise nursing theories as vital tools for structuring and enhancing care quality. To bridge this gap, it is crucial to develop pedagogical strategies that not only make nursing theories more accessible but also demonstrate their relevance and applicability from the earliest stages of training (McEwen and Wills 2022).

The pioneers of nursing science quickly understood the importance of a theoretical foundation in nursing education. They designed training programmes based on a strong theoretical foundation that serves as a reference system for university teaching and contributes to the academisation of the field (Missi et al. 2018). The systematic integration of nursing theories into nursing education programmes not only enhances the value of the education but also facilitates the adoption of theory as a fundamental component of clinical practice (Norris and Newsome 2021). This vision aligns with the idea that teaching disciplinary fundamentals from the first year helps students, who are often receptive and motivated, connect theory to clinical practice, thereby narrowing the gap between these two essential aspects of their learning (Alligood 2021; Meleis 2017). Furthermore, this approach prepares them to apply this knowledge in increasingly complex and dynamic clinical environments (Donohue‐Porter et al. 2017). Conversely, minimising or excluding theoretical content from nursing curricula hinders the acquisition of cognitive skills needed to recognise the theoretical and scientific aspects of nursing practice (Norris and Newsome 2021).

Embedding the theoretical foundations of the discipline in educational programs also plays a crucial role in the dissemination of professional values and the appropriation of nursing knowledge by students (Chinn et al. 2022). A curriculum that integrates these theories and encourages critical reflection is fundamental for the continuous advancement of the profession and for preserving the very existence of the nursing discipline (Smith 2019). Educators, therefore, have the responsibility to promote the development, dissemination and utilisation of nursing knowledge (Thompson and Schwartz Barcott 2019). It is also essential that courses rely on knowledge derived from the nursing discipline to ensure the quality of care and maintain a robust scientific foundation in nursing practice (Smith 2019).

### 1.1 The Review

These considerations clearly highlight the importance of integrating the teaching of nursing theories from the beginning of training. However, it is essential to determine which pedagogical strategies are most effective for teaching these disciplinary fundamentals in initial education. The literature reveals a high degree of creativity and diversity in practices aimed at teaching conceptual models and nursing theories (Risjord 2010). To the best of our knowledge, there is a lack of comprehensive understanding in this area.

### 1.2 Aims

The aim of this scoping review is to: (i) identify existing pedagogical practices for teaching conceptual models and/or theories in nursing science within initial education, (ii) describe the structuring of these teachings, (iii) report on the outcomes of these interventions and (iv) identify the most frequently taught conceptual models or theories in nursing science.

## 2 Methods

To map these elements, a scoping review was conducted following the recommendations of the Joanna Briggs Institute (JBI) (Peters et al. 2024) and the Preferred Reporting Items for Systematic Reviews and Meta‐Analyses Extension for Scoping Reviews (PRISMA‐ScR).

A preliminary search in PROSPERO, MEDLINE, the Cochrane Database of Systematic Reviews, and JBI Evidence Synthesis was conducted. No ongoing systematic review on the topic was identified. The detailed protocol for the scoping review has been published (Demagny‐Warmoes et al. 2024) and registered in the Open Science Framework (OSF) database with the following Digital Object Identifier (DOI): https://osf.io/gj35n.

### 2.1 Search Methods

The databases were searched from their inception to January 2024. The databases PubMed, CINAHL, Education Resources Information Center (ERIC), Web of Science (WOS), and EMBASE were consulted based on three descriptor blocks: ‘nursing theory,’ ‘education, nursing,’ and ‘teaching, methods.’ Additionally, grey literature was explored through Google Scholar, ProQuest, HAL and the Système Universitaire de Documentation (SUDOC).

Sources were exported to Zotero 6.0.27/2023 to identify and remove duplicates, then transferred to the JBI system for unified management, assessment, and information review (JBI SUMARI) (JBI, Adelaide, Australia) (Munn et al. 2019).

### 2.2 Inclusion and Exclusion Criteria

Inclusion and exclusion criteria were used to narrow the research question and select relevant sources (Table 1). The PCC (Participants/Concept/Context) acronym was employed to establish the guiding question (Peters et al. 2024). Here, ‘P’ refers to undergraduate students, ‘C’ to pedagogical/didactic techniques and disciplinary fundamentals, and ‘C’ to nursing education on an international scale. This led to the formulation of the research question: What pedagogical strategies are used to teach conceptual models and/or nursing theories in initial nursing education?

**TABLE 1:** Inclusion criteria and exclusion criteria.

	Inclusion criteria	Exclusion criteria
Participants	Undergraduate students	Graduate students (Master's and PhD) Continuing education
Concept	Explicit pedagogical/didactic practices Conceptual models and/or nursing theories	Undetailed pedagogical/didactic practices Conceptual models and/or theories from other disciplines (e.g., educational sciences) Advocacy for the teaching of disciplinary fundamentals Strategies for integrating disciplinary fundamentals into an institutional curriculum
Context	Nursing education	Other disciplines
Study charactéristics	Journal papers Grey literature Published in French or English Full text available No date restrictions	Books

### 2.3 Search Outcome

**FIGURE 1:**
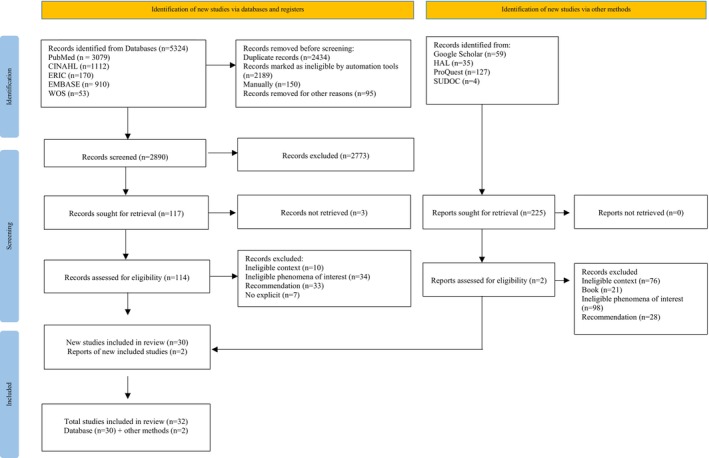
PRISMA flow chart.

The comprehensive database search initially identified 5324 records. After removing duplicates, 2890 records were imported into JBI SUMARI (System for the Unified Management of the Assessment and Review of Information) for title and abstract screening, resulting in 117 articles selected for full‐text review. Of these, three could not be located despite interlibrary loan requests and attempts to contact the authors, reducing the number of articles to 114. After thorough review and conflict resolution, 30 articles were ultimately deemed to meet the inclusion criteria and were retained for extraction. A review of the reference lists of included articles did not reveal any new relevant studies meeting the inclusion criteria, or they were already included. Manual searching added two additional records. In total, 32 articles were included in this scoping review. The search results and study inclusion process are illustrated in the PRISMA flow diagram and detailed in Figure 1.

### 2.4 Quality Appraisal

The selection of titles and abstracts was carried out using the JBI SUMARI tool. This tool enables blind study selection by two reviewers, ensuring reliability and methodological rigour. In cases of persistent disagreement between the two reviewers (ADW—MD) regarding the inclusion of a study, a third evaluator (SC) intervenes to make the final decision.

### 2.5 Data Abstraction and Synthesis

For the extraction and synthesis of full texts, two data‐charting forms were created by author 1 through Microsoft Excel and approved by all coauthors. Relevant characteristics related to the research question were collected in double‐blind (ADW—PQ) using an extraction tool based on the JBI template for sources of evidence details, characteristics, and results extraction, as well as the work of Raynal and Rieunier (2014) on the design of a pedagogical strategy. Once all elements were compiled in narrative form within the tool, results were compared, and in cases of discrepancies, a third author (SC) was consulted to arbitrate.

## 3 Results

### 3.1 Characteristics of Included Articles

This process involves extracting and synthesising key information from each selected article. All included articles (*n* = 32) in this review primarily aim to describe and/or test a pedagogical strategy intended to teach disciplinary fundamentals in initial education. The pedagogical strategies were tested using various methods, including quantitative and qualitative approaches, and occasionally quasi‐experimental designs. However, there is a predominance of pedagogical case reports (81.2%) compared to more robust research methodologies (18.7%) (Table 2).

**TABLE 2:** Characteristics of studies included in the scoping review.

Author(s) year country	Design	Sample	Teaching period	Results
Alderman et al. (2018) Australia	Pedagogical case report	N/A	Final year of the program	The pedagogical strategy used enhances the quality of training and understanding of the fundamentals of care but requires additional resources, extended duration and improved collaboration with clinical departments
Baldwin and Schaffer (1990) USA	Pedagogical case report	46 students	Second year of the program	The pedagogical strategy enhances the understanding of nursing theories, with a perceived usefulness rated by students at 2.8 out of 4 on a Likert scale. However, it requires thorough planning and effective communication among instructors
Batra (1987) USA	Pedagogical case report	30 students	Second year of the program	This pedagogical approach enables students to explore and compare nursing theories, strengthening their understanding of concepts and encouraging creativity. However, it is better suited to groups of fewer than 30 students. Student presentations may lack precision, but these limitations are offset by an early emphasis on nursing identity
Biggs (1996) USA	Pedagogical case report	N/A	N/A	The pedagogical strategy of integrating nursing theories into a computer science course demonstrates that these concepts can be taught in diverse contexts. Students report satisfaction, finding this approach enriching for their understanding and practice, and fostering deeper reflection on nursing care
Bourbonnais and Ross (1985) Canada	Pedagogical case report	N/A	Final year of the program	The pedagogical strategy integrating Neuman's model enabled students to develop critical skills and distinguish nursing issues from medical issues. This structured approach generated student satisfaction, enriching their clinical practice
Caroline (2006) USA	Pedagogical case report	N/A	N/A	The pedagogical strategy combines various techniques to enable students to apply nursing theories to real‐life situations. This approach promotes a diversity of perspectives, and although its effectiveness relies on prior understanding of the theories, it generates genuine enthusiasm among students
Cessario (1987) USA	A quasi‐experimental study	23 students: 14 in the control group and 9 in the experimental group	N/A	The experiment shows that the board game improves scores and makes learning theories more enjoyable. Despite a small sample size, the results suggest that board games enhance learning and motivation, complementing traditional teaching methods
Costello and Barron (2017) USA	Pedagogical case report	N/A	N/A	The pedagogical strategy integrates caritas factors through various methods to raise students' awareness of caring in palliative care. This approach has enhanced their creativity and respect for patient culture
Flanagan and McCausland (2007) USA	Pedagogical case report	140 students	Throughout the program	The pedagogical strategy, adaptable to different group sizes, combines multiple teaching techniques to make nursing theories more accessible. These techniques, considered enjoyable and well‐received by students, require rigorous evaluation
Gramling and Nugent (1998) USA	Pedagogical case report	N/A	First year of the program	The pedagogical strategy for teaching nursing theory, centered on interactive and structured activities, received positive feedback. Although its introduction was considered premature for less experienced students, caring behaviours later emerged during clinical placements and in their written work
Harris (1986) USA	Pedagogical case report	50 students	N/A	The pedagogical strategy initially integrates simplified nursing theory implicitly within courses and clinical placements, before presenting it explicitly in subsequent instruction. This approach leads the majority of students to adopt the model in their practice, reducing resistance to other theories. However, uncertainties remain regarding the continuity of this practice after graduation
Karmels (1993) USA	Pedagogical case report	N/A	N/A	The interactive pedagogical strategy allows students to embody theorists to strengthen their mastery of terminology and understanding of the theorists' perspectives. However, the article does not provide an evaluation of this method's effectiveness or student feedback
Larkin (2018) USA	Pedagogical case report	N/A	Third year of the program	The pedagogical strategy integrates concepts from nursing theories into the narrative of a mindfulness meditation session. This approach aims to cultivate students' awareness of the importance of self‐care while deepening their understanding of theory‐based nursing care in their practice
Levitt and Adelman (2010) USA	Pedagogical case report	N/A	N/A	The pedagogical strategy uses an online role‐playing game, moderated by the instructor, where students embody a nursing theorist and discuss concepts under her identity to deepen their theoretical understanding. This method generated high satisfaction and strong engagement among students
Lohri‐Posey (1999) USA	Pedagogical case report	N/A	Final year of the program	The creative and participatory pedagogical method, such as poster presentations, is effective in enhancing students' understanding of theoretical concepts. This strategy connects abstract theories to clinical practice, making these concepts more concrete and relevant. Students find the activity engaging and feel it fosters critical thinking and collaborative learning
Lowry (1988) USA	Pedagogical case report	N/A	First year of the program	The pedagogical strategy employs a combination of techniques to introduce Neuman's model, enabling students to apply it in both personal and clinical contexts. Students recognise the usefulness of this approach in enhancing their understanding and practice
Maltby et al. (1995) Australia	Pedagogical case report	N/A	First year of the program	The combination of pedagogical techniques effectively integrated the concept of caring among students, demonstrating the approach's effectiveness in grounding this theory from the beginning of their training. However, no evaluation of the strategy or student feedback is provided
Masters (1988) USA	Pedagogical case report	N/A	Second year of the program	The eclectic approach, integrating various theories, strengthened students' ability to link theory with clinical practice. This method fostered critical thinking and problem‐solving, effectively preparing students to apply nursing theories in diverse clinical contexts. Students expressed high satisfaction, highlighting the usefulness of this approach in connecting theoretical knowledge to practical situations
McClure and Gigliotti (2012) USA	Pedagogical case report	N/A	N/A	The creation of a tool titled ‘Medieval Metaphor’ helps students understand and apply Neuman's theory concepts during simulation sessions. This pedagogical approach aids students in grasping the abstract concepts of the theory, stimulates critical thinking and encourages self‐reflection, even without prior training on the theory. While the potential learning benefits are highlighted, no specific data on student satisfaction is provided
Melrose (2006) Canada	Pedagogical case report	N/A	N/A	The informal conversation exercise with theorists was appreciated for its creative and playful approach, enhancing both the understanding of theoretical concepts and clinical decision‐making. Positive feedback confirms its effectiveness in engaging students in an enriching and enjoyable theoretical learning experience
Morales‐Mann and Logan (1990) Canada	Pedagogical case report	50–60 students	First year of the program	The pedagogical strategy, combining multiple approaches to integrate the conceptual model, was enriching for students, despite frustrations related to the gap between academic requirements and clinical practice, as well as the variation in faculty mastery of the model and the lack of familiarity among nursing staff. Despite these obstacles, the method enhanced their theoretical understanding
Murphy (1991) Canada	Pedagogical case report	N/A	Throughout the program	The pedagogical strategy effectively integrates the theoretical model, reinforcing the connection between theory and practice while developing analytical and collaborative skills and professional identity. The absence of formal evaluation makes it challenging to measure the educational impact, but informal feedback indicates high satisfaction, with students appreciating the way concepts were connected to practice
Ross et al. (1987) USA	Pedagogical case report	N/A	Final year of the program	The pedagogical strategy combines various techniques to integrate Neuman's model, enhancing students' ability to analyse and conceptualise a variety of clinical situations. This approach has also improved their understanding of the subjective dimensions of human experience, leading to more relevant assessments of health states. Students provided positive feedback, finding the model useful for addressing complex clinical situations and developing effective intervention strategies
San Martín‐Rodríguez et al. (2020) Spain	Analytical Cross‐Sectional Study	105 students	First year of the program	The pedagogical strategy utilises gamification to teach disciplinary fundamentals, where students must identify an ‘intruder’ among nursing theorists through interactive games. This method has shown positive results, with an average satisfaction score of 7.60 out of 10. Students enjoyed the experience but suggested clarifying expectations and reducing content to avoid cognitive overload
Schuler (2018) USA	Pedagogical case report	N/A	N/A	The arts‐based pedagogical strategy allows students to deepen their understanding of theories by creating artistic representations of them. This approach facilitates the integration of theory into clinical practice, enhances comprehension of concepts, and improves student satisfaction. The author considers arts‐based learning an effective method for teaching complex theoretical concepts in nursing
Sethares and Gramling (2014) USA	Pedagogical case report	9 students	N/A	The pedagogical strategy used to teach nursing theory establishes meaningful relationships between professionals and patients, fostering mutual understanding and collaborative change. Students found this approach relevant to their professional development, although their satisfaction is not explicitly detailed
Singleterry (2020) USA	Analytical Cross‐Sectional Study	37 students	Final year of the program	The pedagogical technique employed has strengthened students' confidence in applying nursing theory in intensive care, particularly regarding the use of technology. Key strengths include a significant improvement in this confidence. While the study does not directly measure student satisfaction, the results demonstrate the effectiveness of simulation in integrating nursing theory into clinical practice
Slaninka (1999) USA	Pedagogical case report	N/A	N/A	The author developed a three‐step pedagogical strategy to encourage student creativity. This innovative method generated high satisfaction among students, who found the assessment fun and engaging. The author concludes that this approach makes learning nursing theories more dynamic and relevant
Voldbjerg et al. (2018) Denmark	Pedagogical case report	160 students	Throughout the program	The pedagogical strategy used has enhanced students' understanding of fundamental care and critical thinking through evidence‐based approaches. Despite initial scepticism from faculty members, the collaboration between the University and regional hospitals facilitated the adoption of the theory. Students provided positive feedback
Webb (1990) England	Pedagogical case report	N/A	Throughout the program	This approach, which combines theory and clinical practice, not only allows students to compare and apply nursing theories but also indirectly raises awareness of these theories in clinical settings by using them in real‐world contexts. Although student satisfaction is not mentioned, the method proves effective as only 3 out of 50 students failed to integrate the model according to the author
Wissmann (1994) USA	Pedagogical case report	N/A	Final year of the program	The pedagogical approach is inspired by an election campaign to teach nursing theories. This method has received positive feedback from students. The author highlights the effectiveness of this approach in developing critical thinking, leadership, communication, and understanding of nursing concepts
Wu et al. (2009) Taiwan	A quasi‐experimental study	40 students in the experimental group	Second year of the program	The study evaluates a strategy combining several pedagogical techniques to improve students' caring competencies. This strategy, using various teaching methods, has significantly enhanced caring behaviours among students. However, it remains challenging to determine which specific method was the most effective

Abbreviation: N/A, not applicable.

**FIGURE 2:**
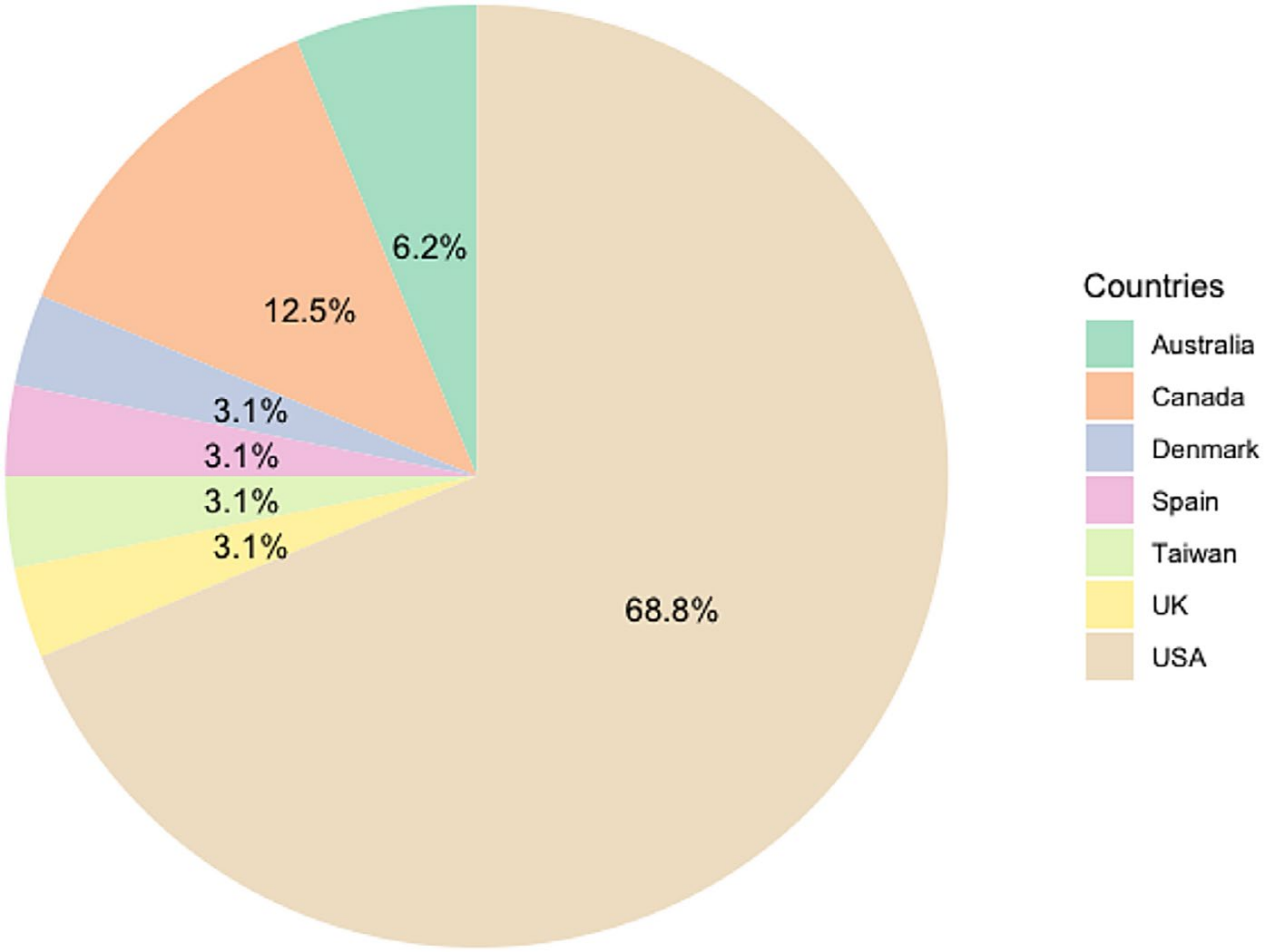
Country characteristics.

This scoping review highlights the international dimension of teaching disciplinary fundamentals, with the majority of contributions from North America (81.2%), followed by Europe (9.3%), Australia (6.2%) and Asia (3.1%). The analysed articles span from 1985 to 2020, with a decadal distribution showing that 18.7% of publications date from 1985 to 1989, 34.3% from the 1990s, 15.6% from the early 2000s and 31.2% after 2011, with a notable increase in publications starting in 2017 (Figure 2).

### 3.2 Sample Characteristics and Implementation Periods

The analysed articles present information on sample sizes and the implementation period of pedagogical strategies for teaching disciplinary fundamentals unevenly. It is important to note that 65.6% of the articles do not include these specific data. Among those that do provide this information, the number of students benefiting from these strategies varies significantly, ranging from nine participants to a cohort of 160 students (Table 2).

Regarding the implementation period of the pedagogical strategies, 40.6% of the articles did not provide this information (*n* = 13). Among the 19 articles that mention the implementation period, the strategies are mostly carried out on a one‐off basis, either during the first year (26.3%) or in the final year (32%). Only 15.7% of the strategies are implemented throughout the academic curriculum, reflecting diverse approaches to integrating disciplinary fundamentals into training programmes (Table 2).

### 3.3 Evaluation of Pedagogical Strategies

The majority of the analysed sources (78.1%) report the results of their pedagogical interventions empirically, often in the form of case studies. These evaluations are mainly based on unstructured observations, informal student feedback, illustrated by verbatims, as well as comments from fellow educators, etc. They primarily focus on understanding and applying theories, student engagement, and highlighting the enjoyable aspects of the strategies. Conversely, only 15.6% of the strategies (*n* = 5) underwent an in‐depth scientific evaluation. Finally, three articles, representing less than 10% of the total, do not present any feedback on the results of their pedagogical intervention, even in empirical form (Table 2).

The quasi‐experimental study by Cessario (1987) involved 23 nursing students, divided into an experimental group of 9 students and a control group of 14 students. The experimental group, which used a specially designed board game for learning nursing theories in addition to standard courses on disciplinary fundamentals, achieved significantly higher post‐test scores than the control group, which only attended the courses (*p* < 0.05). All participants in the experimental group reported that the game motivated and reinforced their learning, and they recommended its permanent inclusion in the course programme.

The quasi‐experimental study by Wu et al. (2009) evaluated the effectiveness of a pedagogical strategy centered on Watson's caring model with 85 female students, divided into an experimental group that volunteered to participate in a 13‐week course based on Watson's conceptual model and a control group that declined the offer. The experimental group showed a significant increase in caring behaviours after the intervention, with an overall mean score rising from 4.21 to 4.44 on the Caring Behaviour Assessment (CBA) scale. This improvement was statistically significant for most subscales, especially for ‘expressing positive/negative feelings,’ with an increase of 0.35 points. The program thus demonstrated its effectiveness and was deemed beneficial for learning about care.

The evaluation of the pedagogical intervention by San Martín‐Rodríguez et al. (2020), using an escape game to teach nursing theories and models to 105 first‐year students, showed positive results. The students achieved a mean score of 8.28 out of 10 for knowledge acquisition and expressed high satisfaction, with an average score of 7.60 out of 10 for the game. Students also recommended the continued use of the game, although 61% suggested adjustments, such as reducing content and improving explanations about game dynamics.

Singleterry (2020) examined the impact of a clinical simulation on 37 students, focusing on the application of Locsin's technological competency as caring in nursing theory. The results showed a significant increase in students' confidence, with gains ranging from 17 to 30 points on a 100‐point scale after the simulation. This method was deemed effective in enhancing confidence and integrating theoretical concepts into clinical practice.

The ongoing pedagogical case study by Baldwin and Schaffer (1990) involved 46 students. The results showed a positive perception of the usefulness of the pedagogical method, with average scores ranging from 2.8 to 3.2 on a four‐point Likert scale (1 indicating minimal usefulness, 4 maximum usefulness). The overall mean score for the method's effectiveness was 3.1. The reliability index of the evaluation, measured by a Cronbach's alpha of 0.94, demonstrated a high internal consistency of responses. This evolving pedagogical case study proved particularly effective in linking theoretical concepts to practice and was well received by students.

### 3.4 Teaching Methods

The majority of the included articles indicate the use of strategies that combine multiple pedagogical techniques (93.8%). Only Cessario (1987) and San Martín‐Rodríguez et al. (2020) presented a single pedagogical technique. A synthesis of the various pedagogical techniques identified for teaching nursing theories is presented in Table 3.

**TABLE 3:** Summary of pedagogical techniques used in the studies included in the scoping review.

Articles	Lecture courses	Reading	Written work	Poster	Case study/care plan	Chart review	Talks	Debate	Interview	Video learning	Role modelling	Role playing	Game	Mindfulness course	Arts learning	Simulation	Laboratory practice	Clinical practice	Practice review
Alderman et al. 2018	x	x														x	x		
Baldwin and Schaffer 1990	x				x												x		
Batra 1987	x	x			x		x	x										x	
Biggs 1996	x		x			x													
Bourbonnais and Ross 1985			x															x	x
Caroline 2006					x		x	x											
Cessario 1987													x						
Costello and Barron 2017		x					x			x				x					x
Flanagan and McCausland 2007	x	x	x		x		x						x						
Gramling and Nugent 1998	x							x	x			x							x
Harris 1986	x	x			x												x	x	
Karmels 1993													x						
Larkin 2018														x					
Levitt and Adelman 2010		x	x					x				x							
Lohri‐Posey 1999		x		x			x												
Lowry 1988	x		x						x										x
Maltby et al. 1995	x	x			x					x									
Masters 1988	x	x	x				x												x
McClure and Gigliotti 2012																x			
Melrose 2006			x					x				x							
Morales‐Mann and Logan 1990	x				x			x										x	
Murphy 1991					x		x	x	x										
Ross et al. 1987	x	x			x													x	x
San Martín‐Rodríguez et al. 2020													x						
Schuler 2018	x	x													x				
Sethares and Gramling 2014									x									x	x
Singleterry 2020																x			
Slaninka 1999		x	x				x												
Voldbjerg et al. 2018	x				x											x			
Webb 1990	x		x		x		x											x	
Wissmann 1994			x				x	x											
Wu et al. 2009					x						x								x

### 3.5 Theoretical Teaching

Theoretical teaching in nursing remains dominated by lectures, which are often enriched with real‐life examples provided by instructors. These lectures, along with recommended readings, represent 46.8% and 40.6%, respectively, of the teaching methods used among the selected articles. Pedagogical readings can include journal articles (Harris 1986; Levitt and Adelman 2010; Ross et al. 1987; Schuler 2018), critical analyses (Flanagan and McCausland 2007), reference books on theories (Batra 1987), or testimonial‐based novels (Costello and Barron 2017). Additionally, students are frequently encouraged to conduct literature searches, notably to create bibliographies on the nursing theories taught (Maltby et al. 1995; Masters 1988; Slaninka 1999). Written assignments take various forms: reflective journals (Lowry 1988), summary sheets (Masters 1988) or memorandums (Wissmann 1994). For more in‐depth analyses, students are encouraged to draft critical essays, where they examine and discuss the theories studied (Bourbonnais and Ross 1985; Slaninka 1999; Webb 1990), while strictly adhering to bibliographic standards (Flanagan and McCausland 2007). These assignments may take the form of typewritten documents during word processing lessons (Biggs 1996) or via online discussion forums (Levitt and Adelman 2010; Melrose 2006).

### 3.6 Practical and Clinical Teaching

The application of nursing theories sometimes takes place in laboratories, where students are encouraged to identify and apply theoretical concepts during practical exercises, such as performing hygiene care on a mannequin (Alderman et al. 2018) or conducting clinical exams (Baldwin and Schaffer 1990). The integration of these theories is also applied in clinical placements (Batra 1987; Bourbonnais and Ross 1985; Morales‐Mann and Logan 1990; Murphy 1991; Ross et al. 1987; Sethares and Gramling 2014; Webb 1990). Instructors play a central role as consultants, facilitators, and models in the application of theoretical concepts (Bourbonnais and Ross 1985).

In the field, instructors oversee the execution of care projects (Morales‐Mann and Logan 1990), data collection (Ross et al. 1987), and conducting interviews aligned with the theoretical perspective being studied (Sethares and Gramling 2014). Some arrangements include the use of clinical placement portfolios specifically designed to assess students' competencies in connection with the taught nursing theory. This approach, in turn, raises awareness among field professionals about nursing theories and their application in clinical practice (Webb 1990).

Finally, although professional practice analyses are often conducted in clinical settings, they can also be carried out in academic settings. These classroom sessions allow for deeper reflection on care, providing a structured framework to revisit and discuss clinical experiences in light of nursing theories. Thus, even outside the clinical context, these discussions offer students the opportunity to fully integrate theoretical concepts into their professional reflection (Bourbonnais and Ross 1985; Costello and Barron 2017; Gramling and Nugent 1998; Lowry 1988; Masters 1988; Ross et al. 1987; Sethares and Gramling 2014).

### 3.7 Simulation and Role‐Playing

Simulation and role‐playing also represent pedagogical opportunities for the practical teaching of nursing theories. Role‐playing allows students to absorb the values and assumptions conveyed by the theory (Levitt and Adelman 2010; Melrose 2006; Wu et al. 2009) while identifying models and counter‐models (Gramling and Nugent 1998). As for simulation, it appears suitable for applying the fundamentals of care (FoC) (Alderman et al. 2018; Voldbjerg et al. 2018), Locsin's theory (Singleterry 2020) or Neuman's theory (McClure and Gigliotti 2012). Furthermore, meditation constitutes an innovative method for teaching nursing theories by integrating mindfulness practice into learning (Costello and Barron 2017; Larkin 2018).

### 3.8 Game‐Based Learning

To make learning more engaging and facilitate the understanding of nursing theories, some authors consider games to be an effective pedagogical strategy, often used as a complement to traditional courses (Flanagan and McCausland 2007). Several initiatives have emerged to teach nursing theories through different types of games. For example, Flanagan and McCausland (2007) developed question‐and‐answer games where students can use their notes or computers to answer questions. Karmels (1993) introduced an innovative approach where students, dressed up as theorists, participate in question‐and‐answer games to help their peers better understand concepts. Meanwhile, Cessario (1987) explored the use of board games as a means of teaching nursing theories. More recently, San Martín‐Rodríguez et al. (2020) created a virtual game using digital resources and based on the American Nurses Association frameworks, offering a modern and interactive approach to teaching nursing theories.

### 3.9 Problem‐Based and Project‐Based Learning

To help students understand how nursing theories apply in real situations, case studies and care projects remain essential pedagogical tools, regardless of the time period or country (Baldwin and Schaffer 1990; Batra 1987; Caroline 2006; Flanagan and McCausland 2007; Harris 1986; Maltby et al. 1995; Murphy 1991; Ross et al. 1987; Voldbjerg et al. 2018; Webb 1990; Wu et al. 2009). Case studies can increase in complexity as the student progresses through their studies (Baldwin and Schaffer 1990; Murphy 1991). Sometimes, cases are presented in video format, making the situations more vivid (Costello and Barron 2017), or are sourced from literature (Maltby et al. 1995). In care projects, data collection may be conducted using electronic patient records (Biggs 1996) or through direct collaboration with patients (Morales‐Mann and Logan 1990). It is also common for a single case to be analysed from different theoretical perspectives, leading to a care project specific to each studied theorist, thus demonstrating to students that various approaches can be applied to the same clinical situation (Batra 1987). This technique illustrates the flexibility of nursing theories in practice (Caroline 2006; Flanagan and McCausland 2007). Case analyses and care projects are often presented orally, either at the clinical site or in the classroom.

### 3.10 Interactive Teaching

Oral presentations also provide students with the opportunity to internalise nursing theories by synthesising and sharing them with their peers during plenary sessions (Batra 1987; Caroline 2006; Costello and Barron 2017; Flanagan and McCausland 2007; Lohri‐Posey 1999; Masters 1988; Murphy 1991; Slaninka 1999; Webb 1990; Wissmann 1994). To make these presentations more dynamic, students adopt creative approaches, such as humorous skits, poems (Murphy 1991) or fictional election campaigns, where they advocate for their favourite theorist for university vice‐president (Wissmann 1994). These oral presentations are sometimes supported by visual tools such as PowerPoint slides (Levitt and Adelman 2010), or posters (Lohri‐Posey 1999; Wissmann 1994) and are followed by debates, which, like interviews (Gramling and Nugent 1998; Maltby et al. 1995), aim to challenge students' beliefs about nursing theories. These discussions allow for the examination of theories, exploration of their application in practice and encouragement of critical reflection (Batra 1987; Caroline 2006; Gramling and Nugent 1998; Levitt and Adelman 2010; Melrose 2006; Morales‐Mann and Logan 1990; Murphy 1991; Wissmann 1994). Exchanges can also take place online in dedicated forums (Caroline 2006; Levitt and Adelman 2010).

### 3.11 Theoretical Foundations in Nursing Education: An Overview of Selected Models and Theories

This literature review identified a total of 27 nursing theories and conceptual models. Among the sources examined, 28% do not explicitly mention the names of the theories taught, merely indicating that their teaching is based on a variety of nursing theories (Caroline 2006; Cessario 1987; Flanagan and McCausland 2007; Levitt and Adelman 2010; Masters 1988; Melrose 2006; San Martín‐Rodríguez et al. 2020; Schuler 2018; Slaninka 1999; Wissmann 1994) (Table 4). Nearly half of the pedagogical interventions recorded focus on a single nursing theory and/or conceptual model (Biggs 1996; Harris 1986; Morales‐Mann and Logan 1990; Murphy 1991; Sethares and Gramling 2014; Singleterry 2020), including those of Neuman (Bourbonnais and Ross 1985; Lowry 1988; McClure and Gigliotti 2012; Ross et al. 1987), Watson (Costello and Barron 2017; Gramling and Nugent 1998; Maltby et al. 1995; Wu et al. 2009) and the FoC (Alderman et al. 2018; Voldbjerg et al. 2018). However, although some articles present a specific pedagogical intervention centred on a particular theory, they also emphasise that other nursing theories and/or conceptual models were taught (Gramling and Nugent 1998; Murphy 1991).

**TABLE 4:**
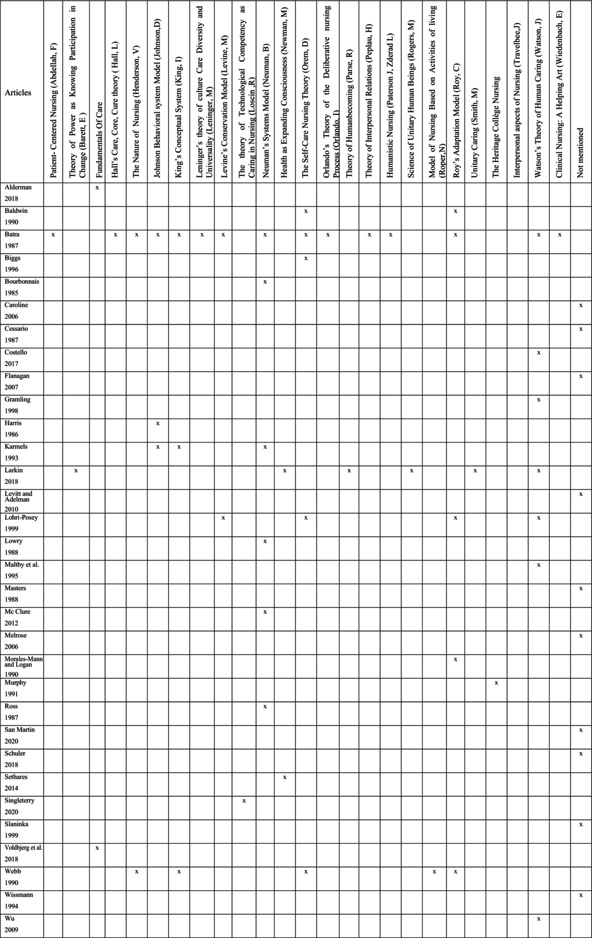
Nursing theories and conceptual models taught in the articles included in the scoping review.

For the second half of the pedagogical interventions, a wide range of conceptual models is employed, spanning from 2 to 15 (Baldwin and Schaffer 1990; Batra 1987; Caroline 2006; Cessario 1987; Flanagan and McCausland 2007; Karmels 1993; Larkin 2018; Levitt and Adelman 2010; Lohri‐Posey 1999; Masters 1988; Melrose 2006; San Martín‐Rodríguez et al. 2020; Schuler 2018; Slaninka 1999; Webb 1990; Wissmann 1994). Watson's conceptual model is the most frequently taught, followed by Neuman, Orem and Roy (Baldwin and Schaffer 1990; Batra 1987; Lohri‐Posey 1999; Morales‐Mann and Logan 1990; Webb 1990). Although Watson's Caring theory has been taught consistently over the decades, the FoC and Loscin theories (Singleterry 2020) are currently gaining popularity.

The majority of articles originate from North America, but none mention the FoC, which is primarily highlighted in Australia and Denmark. Some theories have also been adapted to be more accessible, as seen with the Johnson model (Harris 1986). Heritage College Nursing, for its part, developed its own conceptual model inspired by Roy's model, illustrating the tendency of some nursing schools to develop theoretical frameworks based on existing theories (Murphy 1991).

## 4 Discussion

### 4.1 Discussion and General Conclusions

To our knowledge, this scoping review is the first to systematically analyse the pedagogical methods used to teach disciplinary fundamentals in nursing education. This scoping review highlights North America's leading role in publishing articles on the teaching of fundamental nursing disciplines. This finding is not surprising, as North America is the region where the discipline historically emerged and has particularly flourished (Alligood 2021). Nevertheless, regardless of the region, the selected articles highlight the challenges related to teaching nursing disciplinary fundamentals due to their complexity (Younas and Parsons 2019) and the persistent gap between theory and practice (Lindell 2019). However, the ingenuity of educators in overcoming these obstacles (Risjord 2010) reflects their conviction about the importance of these fundamental knowledge areas in illuminating nursing clinical reasoning (Meleis 2017), conveying professional values (Chinn et al. 2022) and supporting the positioning of nurses in interdisciplinary contexts (Smith 2019). The reviewed articles thus emphasise the necessity of integrating these fundamentals from the beginning of education so that students can naturally internalise nursing theories and effectively utilise them in clinical practice (McEwen and Wills 2022; Norris and Newsome 2021).

The mapping of pedagogical strategies used to teach nursing disciplinary fundamentals, conducted within this scoping review, reveals that educators adopt a multimodal approach, combining various techniques to fully leverage different learning channels. These techniques include traditional lectures, simulation, artistic approaches, virtual games, meditation, etc. This diversity in pedagogical approaches appears essential in overcoming students' reluctance, as they may sometimes perceive these courses as boring or fail to immediately grasp the relevance of nursing theories and/or conceptual models (Bourbonnais and Ross 1985; Harris 1986; Lohri‐Posey 1999; Murphy 1991; Slaninka 1999). Moreover, these active pedagogical methods aim to meet the needs of new generations of learners by accommodating their varied learning styles, well‐being and current educational innovations (Kavanagh and Sharpnack 2021). This multimodal approach also aims to strengthen student engagement and knowledge retention (Cessario 1987; Levitt and Adelman 2010; Lohri‐Posey 1999; Melrose 2006; Slaninka 1999; Webb 1990). However, accurately evaluating the effectiveness of these multimodal approaches sometimes presents a challenge, as it can be difficult to determine which instructional technique had the most significant impact on student learning (Wu et al. 2009).

The scoping review highlights that traditional lectures remain an essential component of nursing education, while new pedagogical approaches incorporating modern technologies, such as simulation and virtual games, are particularly well suited for teaching nursing theories. Moreover, the active involvement of instructors and their expertise in the subject matter is crucial in bridging the gap between theory and practice (Morales‐Mann and Logan 1990; Voldbjerg et al. 2018). In this regard, professional practice analyses based on nursing theories and conducted in a tripartite manner between instructors, students and clinicians represent a promising lever to enhance the dissemination of these theories and raise clinical teams' awareness of situating their practice within an epistemological and ontological framework (Meleis 2017).

Most articles indicate that nursing education programmes tend to integrate multiple theories and/or conceptual models, either simultaneously or progressively, rather than focusing exclusively on a single theory or conceptual model. This approach appears to allow students to compare the various perspectives of theorists, thereby enriching their understanding of care and illustrating the flexibility of nursing theories in practice (Caroline 2006; Flanagan and McCausland 2007; Wissmann 1994). Interestingly, some educational programmes adapt existing conceptual models to suit their specific contexts or create new ones based on these theoretical foundations. Finally, the emergence of innovative theories such as the FoC has generated growing interest among educators due to their potential to enrich nursing practice and address contemporary challenges (Alderman et al. 2018; Voldbjerg et al. 2018). Despite this evolution, established theories, such as Watson's, remain relevant due to ongoing research that enriches and adapts them to the current context (Sitzman and Watson 2018). However, it is noteworthy that no specific pedagogical technique has been identified for teaching middle‐range theories, despite their reputation for being more easily understood (Meleis 2017).

For these pedagogical initiatives to be fully successful, collaboration with all stakeholders and strong institutional support are essential (Voldbjerg et al. 2018). When institutional projects are based on a nursing theory or conceptual model, they help guide practice consistently across all subjects and permeate the entire educational program (Alderman et al. 2018; Bourbonnais and Ross 1985; Gramling and Nugent 1998; Harris 1986; Lohri‐Posey 1999; Lowry 1988; Morales‐Mann and Logan 1990; Murphy 1991; Ross et al. 1987; San Martín‐Rodríguez et al. 2020; Voldbjerg et al. 2018; Webb 1990; Wissmann 1994; Wu et al. 2009). However, even in the absence of institutional support or when disciplinary fundamentals are not integrated into the educational program, the motivation and commitment of instructors still lead them to find ways to raise students' awareness (Biggs 1996).

Many articles focus on pedagogical case studies, often based on unstructured observations and informal student feedback. Although this feedback constitutes a form of empirical evaluation, it limits the generalisation of results to other contexts. Similarly, the lack of homogeneity in pedagogical techniques and samples, when reported, complicates comparisons between studies and highlights the importance of methodological transparency to strengthen the validity of conclusions. Regarding the quasi‐experimental study by Wu et al. (2009), it would also have been pertinent to conduct a pre‐test a few months after the intervention to assess whether the acquired knowledge is durable or volatile, thus enabling a better empirical evaluation of the long‐term effects of pedagogical interventions. Finally, many articles report an improvement in student engagement due to the implemented pedagogical strategy, but without specifying the exact nature of this engagement. The multidimensionality of this concept and the diversity of possible contexts complicate its interpretation. Furthermore, the evaluation of student engagement rarely relies on psychometrically validated scales, thus limiting the reliability of the results reported in these studies.

Nonetheless, recent articles (Alderman et al. 2018; San Martín‐Rodríguez et al. 2020; Singleterry 2020; Voldbjerg et al. 2018) show a promising trend toward more robust research methods or consider such approaches for the future. In the current context of evidence‐based education, it would be beneficial to experiment with robust pedagogical interventions for teaching disciplinary fundamentals. These empirically tested practices could not only validate the effectiveness of pedagogical strategies but also ensure their applicability in diverse contexts.

### 4.2 Implications for Nursing Practice and Research

The chronological analysis reveals a relatively low number of publications between 2000 and 2010, which could be related to the rise of evidence‐based practice, leading to a shift in educational and clinical priorities. This observation is echoed by Parse (2016), who wonders, ‘Where Have All the Nursing Theories Gone?’ During this period, the emphasis on other priorities in educational programs sometimes resulted in a reduction of time dedicated to teaching disciplinary theories. However, certain initiatives, such as that of Biggs (1996), demonstrate that creative solutions, like utilising computer classes, can be implemented to integrate these theories despite educational constraints. Since 2015, a resurgence in publications has been observed in the scoping review, likely in response to a reassessment of the importance of nursing theories in clinical practice, especially in light of global challenges such as the COVID‐19 pandemic (Fernandes and da Silva 2020). This trend also reflects an increased awareness of the need to maintain a strong theoretical foundation to preserve the unique identity of nursing science, particularly in increasingly common interprofessional contexts (Alligood 2021; Chinn et al. 2022). Additionally, growing institutional support aims to educate new generations on these fundamentals to ensure the sustainability of the discipline (Jones et al. 2023; National League for Nursing 2024). Finally, the rise of middle‐range theories has strengthened the clinical relevance of theoretical education (Im and Ju Chang 2012).

This scoping review aligns with the current trend toward strengthening the teaching of disciplinary fundamentals in nursing science. By mapping pedagogical practices, it not only provides a global perspective on the teaching of disciplinary fundamentals internationally but also serves as a source of inspiration and a foundation for developing courses specific to disciplinary fundamentals, a field often perceived as complex and intimidating for educators. In the era of evidence‐based teaching and learning, it is essential to evaluate pedagogical strategies for teaching these fundamentals in order to encourage their integration into curricula.

### 4.3 Strengths and Limitations

The strengths of this scoping review include a systematic and comprehensive search in databases, conducted according to a rigorously published protocol, which enhances the transparency of the process. The authors independently assessed eligibility and extracted data, thereby ensuring the reliability of the results. Furthermore, the data were analysed and discussed by the entire research team, strengthening the credibility and intersubjectivity of the study. The use of a recognised methodological framework also provided methodological robustness to the scoping review.

This scoping review also has limitations. First, the search was limited to the PubMed, CINAHL, ERIC, WOS and EMBASE databases, as well as Google Scholar, HAL, ProQuest and SUDOC for grey literature. Another major limitation is the restriction of the scoping review to sources published in French and English. The conclusions of the scoping review are also affected by the heterogeneity of pedagogical interventions, student samples and sometimes insufficiently detailed methodologies. Despite these limitations, the subject remains crucial for programmes aiming to strengthen nursing identity by promoting disciplinary fundamentals.

## 5 Conclusion

The results of this scoping review reveal a wide diversity of pedagogical techniques, ranging from traditional methods to creative and innovative approaches, reflecting ongoing efforts to make the teaching of nursing theories and conceptual models more relevant and accessible. These theories and conceptual models remain pillars of the discipline, and interest in their teaching has significantly increased in recent years across all continents.

However, to better understand the impact of these pedagogical strategies on student engagement, more rigorous research is needed. Additionally, it would be beneficial to explore new pedagogical approaches, incorporating cultural and artistic practices such as debate–theatre, to assess their potential to stimulate students' cognitive engagement in learning disciplinary fundamentals. In sum, the mapping of pedagogical techniques conducted in this review provides valuable support for educators, offering them a structured resource to diversify and enrich the teaching of nursing theories.

## Author Contributions

Made substantial contributions to conception and design, or acquisition of data, or analysis and interpretation of data: A.D.‐W., M.D., P.Q., S.C. Involved in drafting the manuscript or revising it critically for important intellectual content: A.D.‐W., P.Q., S.C. Given final approval of the version to be published. Each author should have participated sufficiently in the work to take public responsibility for appropriate portions of the content; A.D.‐W., P.Q., S.C. Agreed to be accountable for all aspects of the work in ensuring that questions related to the accuracy or integrity of any part of the work are appropriately investigated and resolved. A.D.‐W., P.Q., S.C.

## Conflicts of Interest

The authors declare no conflicts of interest.

## Supporting information


**Data S1.** Supporting Information.

## Data Availability

The data is available and there are no other articles using the same dataset.
